# Functional Expression, Purification and Identification of Interaction Partners of PACRG

**DOI:** 10.3390/molecules26082308

**Published:** 2021-04-16

**Authors:** Tiankai Liu, Haizhou Zhao, Shaofen Jian, Shu Gong, Sainan Li, Yanhong Ma, Jun Chen, Wenhua Liu

**Affiliations:** 1 School of Life Sciences, Zhaoqing University, Zhaoqing 526061, China; 16tkliu@alumni.stu.edu.cn (T.L.); zhaohaizhou012@163.com (H.Z.); jianshaofen95@163.com (S.J.); Gongshu@zqu.edu.cn (S.G.); lisainan2001@sina.com.cn (S.L.); mayh6@mail2.sysu.edu.cn (Y.M.); 2 Department of Chemistry, City University of Hong Kong, Hong Kong 999077, China; 3 Department of Pharmacognosy, School of Traditional Chinese Pharmacy, China Pharmaceutical University, No. 24 Tongjia Lane, Nanjing 210009, China

**Keywords:** PACRG, cold-shock vectors, recombinant expression, purification, interaction partners

## Abstract

*PACRG* (*Parkin* co-regulated gene) shares a bi-directional promoter with the Parkinson’s disease-associated gene *Parkin*, but the physiological roles of PACRG have not yet been fully elucidated. Recombinant expression methods are indispensable for protein structural and functional studies. In this study, the coding region of *PACRG* was cloned to a conventional vector pQE80L, as well as two cold-shock vectors pCold II and pCold-GST, respectively. The constructs were transformed into *Escherichia coli* (DE3), and the target proteins were overexpressed. The results showed that the cold-shock vectors are more suitable for PACRG expression. The soluble recombinant proteins were purified with Ni^2+^ chelating column, glutathione S-transferase (GST) affinity chromatography and gel filtration. His_6_ pull down assay and LC-MS/MS were carried out for identification of PACRG-binding proteins in HEK293T cell lysates, and a total number of 74 proteins were identified as potential interaction partners of PACRG. GO (Gene ontology) enrichment analysis (FunRich) of the 74 proteins revealed multiple molecular functions and biological processes. The highest proportion of the 74 proteins functioned as transcription regulator and transcription factor activity, suggesting that PACRG may play important roles in regulation of gene transcription.

## 1. Introduction

*PACRG* (*Parkin* co-regulated gene) was cloned and identified in 2003 [1]. *PACRG* is approximately 600 kb in length and located on the antisense strain of the *Parkin* gene with a head-to-head arrangement. The two genes shares a bi-directional promoter and their transcription starting points are only 204 bp away. The open reading frame (ORF) of *PACRG* encodes 257 amino acids with a molecular weight of about 30 kD. PACRG is highly conserved among various organisms, but no functional domain of PACRG has been identified yet. Parkin is an E3 ubiquitin ligase, and mutation or deletion of the *Parkin* gene is associated with autosomal recessive juvenile Parkinson’s disease (ARJP) [2].

Previous studies reported that PACRG was capable of stabilizing microtubule structure via binding with α, β-tubulin [3]. Deletion of PACRG caused a movement defect of flagellum and cilium, and this may be attributed to the factor that PACRG controls axonemal dynein-drive microtubule sliding [4,5]. Loss of PACRG led to male sterility in mice due to sperm motility deficiency [6]. These results described above implicate that PACRG plays an important role in microtubule-mediated movement [7]. In addition, PACRG is a component of Lewy bodies, and it is able to decrease the cell death induced by Pael-R which is a substrate of Parkin, suggesting the involvement of PACRG in neurodegeneration pathogenesis [8,9]. Moreover, variants in the bi-directional promoter region shared by *PACRG* and *Parkin* are associated with susceptibility to several infectious diseases, such as leprosy, tuberculosis, typhoid and paratyphoid fever, as well as leukemia and human astrocytic tumors [10,11,12,13]. Recently, Meschede et al. reported that PACRG protects against TNF-induced cell apoptosis, and this result may help to explain the association of *PACRG* and *Parkin* polymorphisms with susceptibility to these infectious diseases [14].

Until now, physiological functions and interaction partners of PACRG have been poorly elucidated. Under this situation, purified recombinant PACRG is indispensable for investigating its physiological roles. An *E. coli* prokaryotic expression system offers many advantages: simple culturing conditions, rapid bacterial growth, relative high-level expression and low cost. PACRG is easily degraded in eukaryotic cells [9], suggesting that PACRG is probably a hard-to-express protein. Li et al. first observed that expression of PACRG alone showed weak signal, while co-expression of MEIG1 with PACRG led to obvious enhancements of the protein levels in *E. coli* cells, indicating MEIG1 is able to stabilize PACRG [15]. Khan et al. reported a similar co-expression profile, and they first solved the structure of the PACRG/MEIG1 complex using the co-expression approach [16]. Additionally, their results showed that expression of GST-fused PACRG formed inclusion bodies by using the pGEX-6p1 vector [16]. These results confirm that it is difficult to achieve soluble and effective expression of PACRG. In this study, we attempt to develop a facile approach for soluble expression of PACRG by using a conventional vector pQE80L, as well as two cold-shock vectors, pCold II and pCold-GST. Furthermore, we used the purified recombinant proteins to identify interaction partners of PACRG in HEK293T (Human embryonic kidney 293T) cell lysates, and this work could facilitate the understanding of the physiological roles of PACRG.

## 2. Results

### 2.1. Recombinant Expression of PACRG

**Figure 1:**
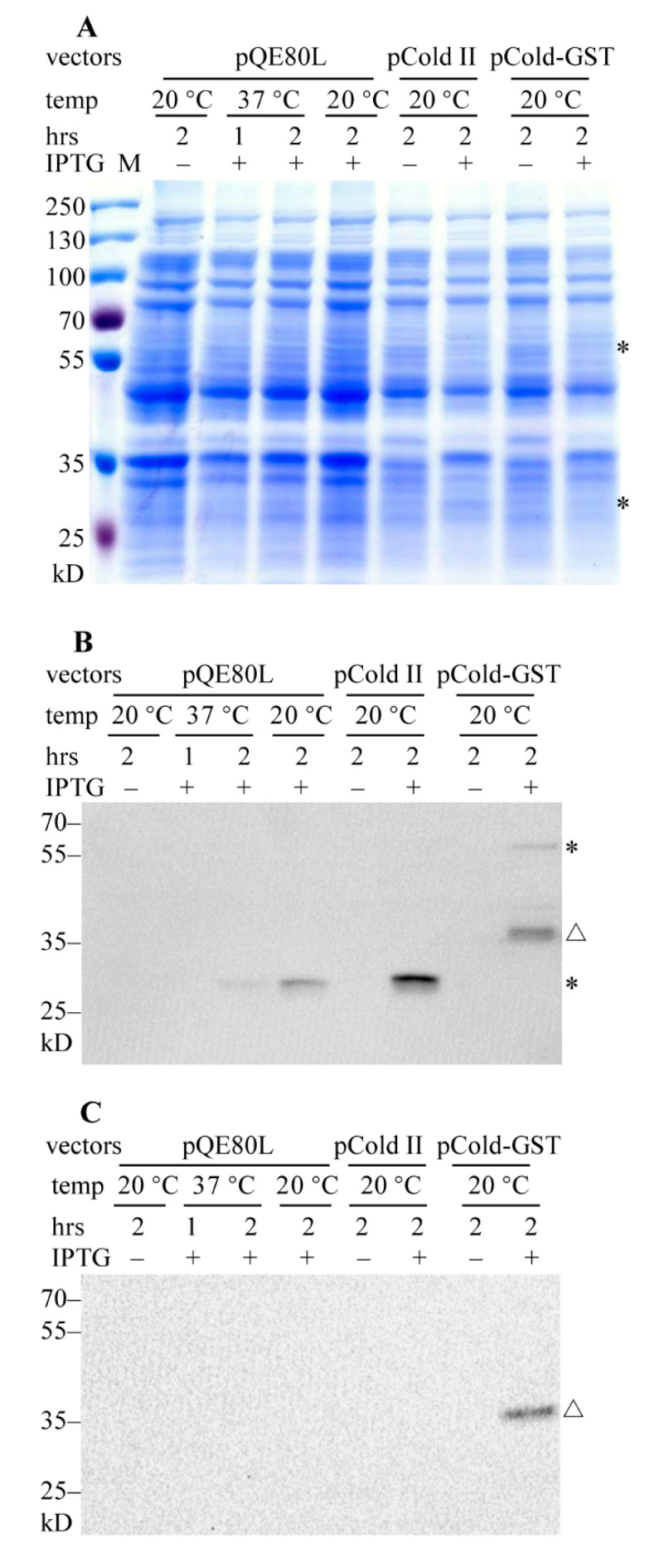
SDS-PAGE analysis of the expressions of recombinant PACRG (*Parkin* co-regulated gene). (**A**) Supernatant fractions of the cell lysates were carried on SDS-PAGE and Coomassie brilliant blue (CBB) staining. * denotes the expected migration location of ~60 kD His_6_-GST-PACRG (upper) and ~31 kD His_6_-PACRG (lower); M, protein marker (Thermo). (**B**) WB analysis of recombinant PACRG in supernatant fractions of the cell lysates with PACRG Ab (Abcam). * denotes His_6_-GST-PACRG (upper) and His_6_-PACRG (lower); △ denotes degraded His_6_-GST-PACRG. (**C**) WB analysis of recombinant PACRG in pellet fractions of the cell lysates with the same Ab. △ denotes ~38 kD degraded His_6_-GST-PACRG. ~30 µg protein was loaded for each sample.

Expression of pQE-PACRG was induced with 0.5 mM isopropyl-D-thiogalactopyranoside (IPTG) for 1 h and 2 h at 37 °C, and for 2 h at 20 °C; while pCold-PACRG and pCold-GST-PACRG were induced with 0.5 mM IPTG for 2 h at 20 °C. The supernatants of the cell lysates were subjected to SDS-PAGE and Coomassie brilliant blue (CBB) staining. However, no apparent overproduction band of His_6_-PACRG (~31 kD) or His_6_-GST-PACRG (~60 kD) could be discriminated in corresponding lanes in the gel (Figure 1A). These results suggested that expressions of PACRG in the three vectors are too weak to be observed by CBB staining.

We further detected the expressions of PACRG with western blot (WB). As shown in Figure 1B, recombinant PACRG was observed in the supernatant fractions of induced *E. coli* cell lysates. Plasmid pQE-PACRG showed a relative weak expression at 20 °C or 37 °C for 2 h, but not at 37 °C for 1 h. pCold-PACRG indicated an obvious expression of His_6_-PACRG at 20 °C for 2 h. While pCold-GST-PACRG showed a ~60 kD His_6_-GST-PACRG band, and several degraded fragments, among them a major ~38 kD degraded band. These data indicate that cold-shock vectors have advantages over pQE80L vector in PACRG overproduction.

We also measured the overproduction of PACRG in the pellet fractions of the cell lysates with WB. As shown in Figure 1C, no His_6_-PACRG or His_6_-GST-PACRG band is visible in the pellet fractions, except for a ~38 kD degraded His_6_-GST-PACRG band. These results suggest that the majorities of His_6_-PACRG and His_6_-GST-PACRG are soluble.

The cold-shock vector system has advantages for high-level expression of target protein under low temperature conditions, with increased solubility and minimal background protein production [17]. When the cultivation temperature is quickly dropped to 15 °C, cold-shock promoter such as CspA is strongly activated, resulting in a marked expression of cold-shock proteins. At the same time, growth of *E. coli* cells and synthesis of the host proteins are temporarily suppressed. Our results confirm that the cold-shock vectors are more appropriate than the conventional pQE80L vector for PACRG overexpression.

Synthesis of the N-terminal His_6_-fused target protein will be terminated due to lack of histidine in the medium [18]. To ask if this is the putative reason for the weak expression of recombinant PACRG, we utilized the control vector pCold-GST, which also containing a His_6_ tag, to induce overproduction with 0.5 mM IPTG for 2 h at 20 °C. High-level expression of GST was observed in CBB staining (Figure S1). These results indicate that both the bacterial system and the medium are appropriate for expression of these vectors.

### 2.2. Optimizations of Expression Conditions of pCold-PACRG and pCold-GST-PACRG

**Figure 2:**
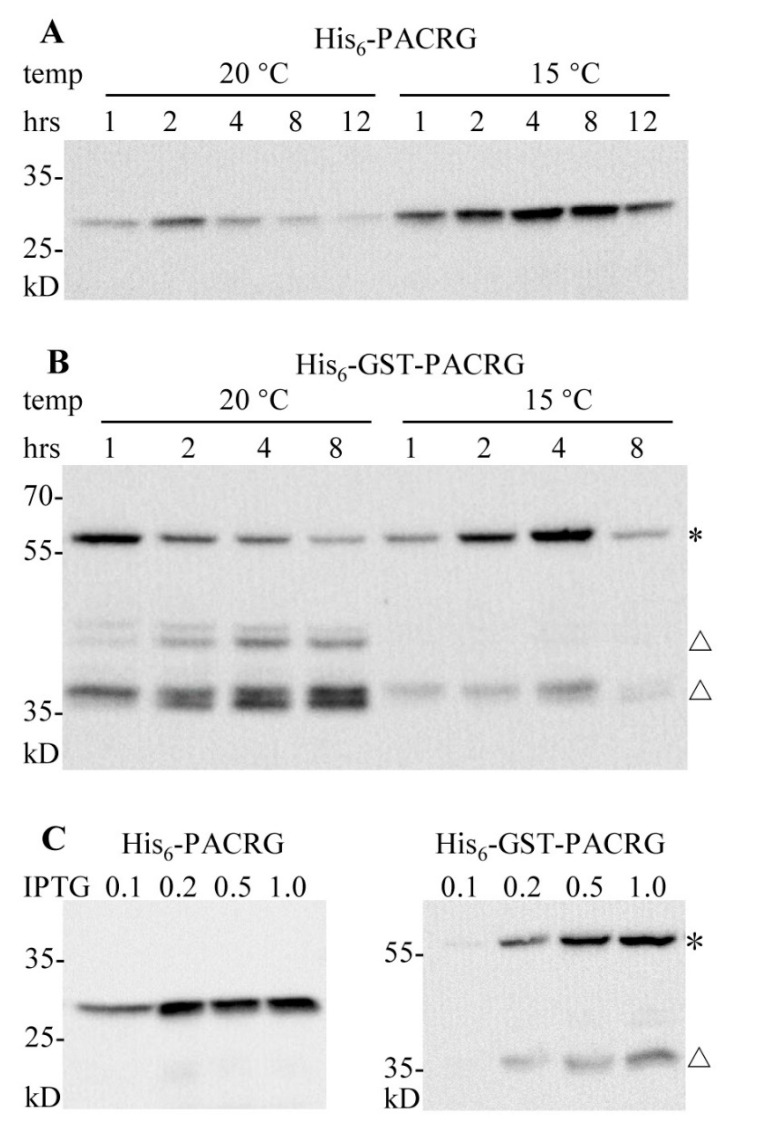
Optimization of expression conditions for pCold-PACRG and pCold-GST-PACRG. (**A**,**B**) WB analysis of pCold-PACRG and pCold-GST-PACRG expressions with 0.5 mM IPTG for various induction durations at 20 °C and 15 °C, respectively. (**C**) WB analysis of pCold-PACRG and pCold-GST-PACRG expressions induced with various concentrations of IPTG for 4 h at 15 °C. * denotes His_6_-GST-PACRG; △ denotes degraded His_6_-GST-PACRG.

Next, we tested whether a lower temperature 15 °C would benefit the expressions. Induction expression of pCold-PACRG was performed with 0.5 mM IPTG for different durations at 20 °C and 15 °C, and the supernatant fractions were subjected to WB. When using 20 °C as growth temperature, the peak expression level appeared at 2 h of post induction (Figure 2A). At 15 °C cultivation temperature, the maximum expression yields were observed at 4 h and 8 h of induction time. The optimal expression conditions for pCold-PACRG were found as cultivation at 15 °C for 4 h or 8 h induction when using 0.5 mM IPTG (Figure 2A).

Expression profiles of pCold-GST-PACRG were showed in Figure 2B. The ~60 kD full-length His_6_-GST-PACRG bands, along with several smaller degraded bands (~45 kD and ~38 kD) were observed. The highest yields of full-length His_6_-GST-PACRG were produced at 20 °C after 1 h of induction, and at 15 °C for 4 h of induction. The degraded bands appeared mainly in the fractions of cultural cells at 20 °C. The optimal expression parameters for pCold-GST-PACRG were found as cultivation at 15 °C for 4 h induction when using 0.5 mM IPTG. Based on these results, lower temperature 15 °C was more suitable for the expressions of the two vectors, and the peak yields of the two vectors showed no obvious difference (Figure 2A,B).

IPTG induction concentrations for the two plasmids were further investigated. As shown in Figure 2C, 0.2, 0.5 and 1.0 mM IPTG induced high yields for pCold-PACRG, while 0.5 and 1.0 mM IPTG were optimal concentrations for pCold-GST-PACRG. Thus, we utilized 0.5 mM IPTG for the next experiments.

### 2.3. Purifications of the Recombinant Proteins

**Figure 3:**
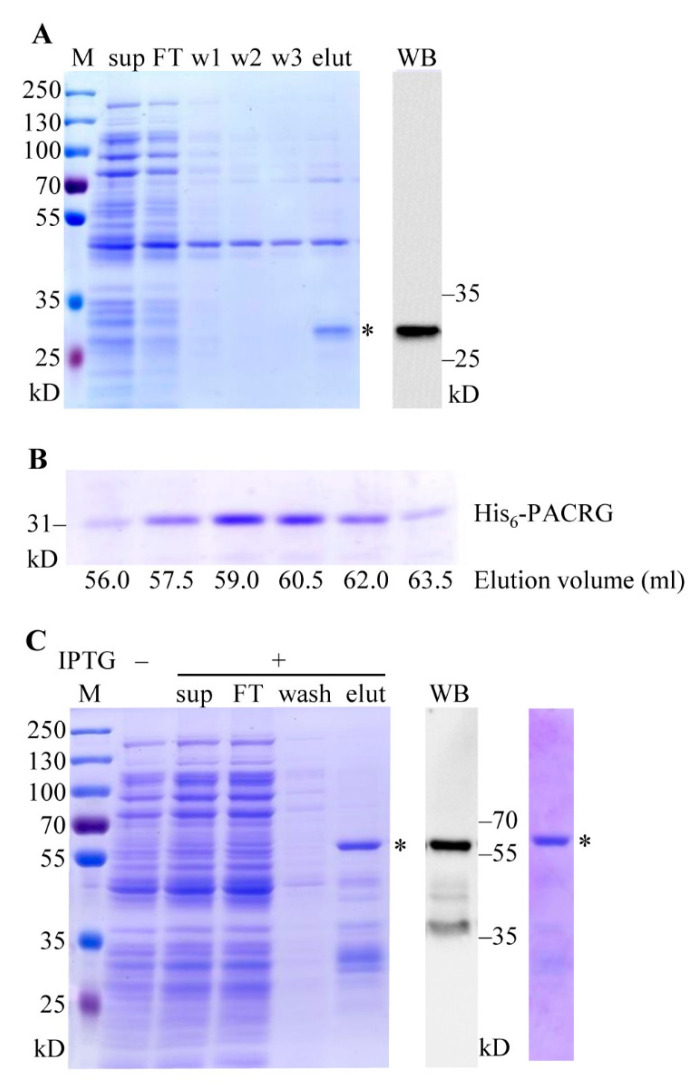
Purifications of His_6_-PACRG and His_6_-GST-PACRG. (**A**) Purification of pCold-PACRG expression product. Left panel: SDS-PAGE analysis and CBB staining of Ni^2+^ chelating purification fractions. Sup, supernatant; FT, flow through; w 1–3, fractions washed with sonication buffer, 2 mM and 5 mM imidazole, respectively; elut, fraction eluted with 50 mM imidazole. Right panel: WB analysis of the eluted fraction with PACRG Ab. * denotes His_6_-PACRG. (**B**) Purification of His_6_-PACRG by gel filtration. 4 mL of Ni^2+^-charge purified His_6_-PACRG was loaded on the Sephacryl™ S-200 column, and eluted with the working buffer. The fractions were collected at 1.5 mL/tube manually, and detected with SDS-PAGE and CBB staining. (**C**) Purification of pCold-GST-PACRG expression product. Left panel: CBB staining of GST affinity purification fractions. Sup, supernatant; FT, flow through; wash, fraction washed with sonication buffer; elut, fraction eluted with 20 mM GSH. Middle panel: WB analysis of the eluted fraction with PACRG Ab. Right panel: CBB staining of gel filtration fraction. * denote His_6_-GST-PACRG. M, protein marker.

Expression products of pCold-PACRG were purified using two steps procedures involving Ni^2+^ chelating purification and gel filtration. As shown in Figure 3A (left and right panel), the expected ~31 kD His_6_-PACRG was eluted with 50 mM imidazole, and confirmed by WB. After preliminary purification, the eluted fraction was concentrated and subsequently applied to gel filtration. The results showed a normal eluting profile for His_6_-PACRG, and the elution volume of the peak fraction was ~59 mL (Figure 3B). In addition, our preliminary data indicated that the void volume of the column detected with blue dextran was ~40 mL, and the elution volumes of BSA (66.4 kD) and cytochrome c (12.5 kD) were ~51 mL and ~68 mL, respectively. The elution volume of His_6_-PACRG (~59 mL) laid in between the ones of BSA (~51 mL) and cytochrome c (~68 mL), suggesting that the estimated molecule weight of the purified His_6_-PACRG matched its theoretical molecule weight 31 kD. These results indicated that His_6_-PACRG was a monomer in the elution fractions, and supported the conclusion that His_6_-PACRG was properly folded, to some content. The final yield of the purified His_6_-PACRG was established at ~1.0 mg/L of Luria-Bertani (LB) culture medium.

The recombinant His_6_-GST-PACRG was purified with GST affinity purification, followed by gel filtration. The ~60 kD full-length His_6_-GST-PACRG, along with various smaller size proteins, were eluted with 20 mM GSH (Figure 3B, left panel). The recombinant His_6_-GST-PACRG was verified with WB (Figure 3B, middle panel), and further purified with gel filtration (Figure 3B, right panel). The elution volume of His_6_-GST-PACRG was ~53 mL, suggesting that the purified His_6_-GST-PACRG was a monomer in the elution fraction. Final yield of the protein was ~1.5 mg/L of LB culture medium.

### 2.4. Enriched Analysis of the PACRG Interaction Partners

**Figure 4:**
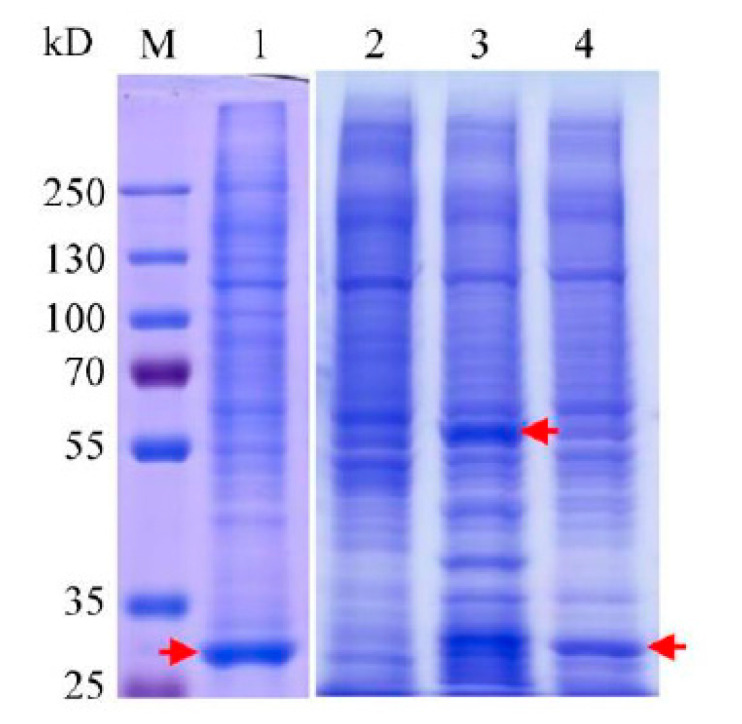
SDS-PAGE analysis of pull down assays using His_6_-PACRG and His_6_-GST-PACRG as baits. Lane 1: pull down assay using His_6_-GST as bait. Arrow indicates the position of His_6_-GST. Lane 2: pull down assay of Ni^2+^-charged resin. Lane 3: pull down assay using His_6_-GST-PACRG as bait. Arrow indicates the position of His_6_-GST-PACRG. Lane 4: pull down assay using His_6_-PACRG as bait. Arrow indicates the position of His_6_-PACRG.

To detect PACRG-binding proteins in HEK293T cell lysates, pull down assays were carried out by using purified His_6_-PACRG and His_6_-GST-PACRG as baits. The total proteins bound to the baits, or the Ni^2+^-charged resin and His_6_-GST were analyzed with SDS-PAGE (Figure 4), and identified by LC-MS/MS. Bound proteins of Ni^2+^-charged resin and purified His_6_-GST were used as background subtraction for His_6_-PACRG and His_6_-GST-PACRG, respectively. A total number of 74 proteins in the two experiments were identified as PACRG interaction partners (Table S1). According to the *p* value < 0.05, gene ontology (GO) enrichment analysis (FunRich) of the 74 proteins revealed multiple molecular functions, including transcription factor activity containing ten proteins, transcription regulator activity consisting of eight proteins, along with other ten classifications of functions each of which contained one to five proteins, respectively (Table 1).

Analysis of the 74 proteins disclosed diverse types of significantly enriched biological processes, including regulation of nucleotide metabolism containing 26 proteins, protein metabolism consisting of nine proteins, cell proliferation with two proteins (Table 2). Beyond that, nuclear organization and biogenesis, neurotransmitter transport, amino acid and derivative metabolism, cell surface receptor linked signal transduction, regulation of immune response and regulation of translation, each of which contained one protein, were also identified as significant biological processes (Table 2).

**Figure 5:**
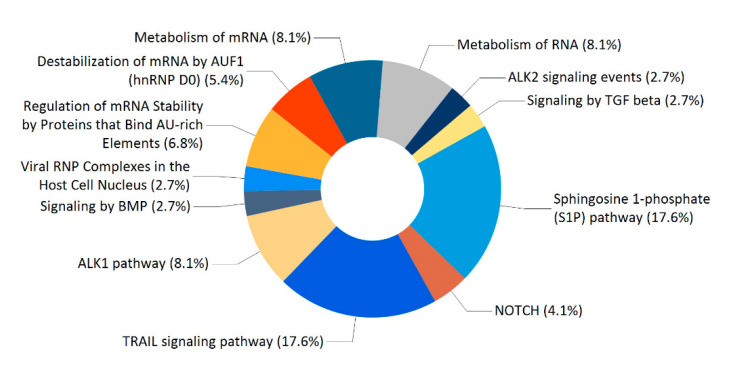
Classifications of biological pathways for the 74 proteins.

Enrichment analysis of the 74 proteins revealed a large number of significant biological pathways. Figure 5 showed the 12 biological pathways with the lowest *p* values. The four main ones include sphingosine 1-phosphate (S1P) pathway, TRAIL signaling pathway, metabolism of mRNA, and ALK1 pathway.

## 3. Discussion

In the present study we performed the recombinant expressions of PACRG using a conventional vector and two cold-shock vectors pCold II and pCold-GST. Our results showed that the cold-shock vectors are more suitable for PACRG expression, and the majorities of the recombinant PACRG were soluble. Moreover, a lower temperature of 15 °C gave higher yields, suggesting that this result may be attributed to inhibition degradation of the recombinant proteins in the host bacteria at the lower temperature (Figure 2). Previous investigation indicated that recombinant expression of PACRG alone resulted in little or no signal, while expression of GFP-fused PACRG formed inclusion bodies [16]. The pCold-GST vector was developed for improving expression of low solubility proteins and unstable proteins, and it has been successfully applied for expressions of multiple proteins [19,20]. Our data showed that the majority of the recombinant His_6_-GST-PACRG was soluble by using the vector pCold-GST, confirming that the cold shock vector could increase the solubility of recombinant proteins. After induction expressions, the recombinant His_6_-PACRG and His_6_-GST-PACRG were further purified with Ni^2+^ chelating column, GST affinity chromatography and gel filtration, and the two purified proteins were obtained at yield levels of ~1.0 mg/L and ~1.5 mg/L of LB culture medium, respectively. The yields were not high, but at a reasonable level. In addition, our data indicated that His_6_-GST-PACRG tended to degrade to smaller fragments, especially at 20 °C growth condition (Figure 2B). GST itself expressed with pCold-GST was stable until 12 h post induction at 20 °C (Figure S1), so the degradation of His_6_-GST-PACRG should be due to cleavages of PACRG. Based on the molecular weight calculations, the ~38 kD degraded His_6_-GST-PACRG should contain a ~30 kD His_6_-GST, and a ~8 kD N-terminal fragment of PACRG which is a divergent region in PACRG orthologs [3]. Moreover, the ~38 kD degraded His_6_-GST-PACRG tended to be insoluble in the lysate (Figure 1C). Considering that PACRG is an aggregated component of Lewy bodies [8], the question of whether the N-terminal divergent region of PACRG is responsible for its aggregation in Lewy bodies may deserve to be further investigated.

Pull down assay and LC-MS/MS identified a total of 74 potential interaction partners of PACRG, including several identified previously interactors, namely HSPA1A, HSPA8 and TUBB2B, which is a member of TUBB isforms (Table S1) [8]. Enrichment analysis showed that a higher proportion of the PACRG interaction partners functioned as transcription regulator and transcription factor activity, suggesting that PACRG may play an important role in regulation of gene transcription (Table 1). Transcriptional regulation activities of PACRG have been reported previously in studies in which PACRG promoted nuclear factor κB (NF-κB) activation [14], and acted upstream of the transcription factor DAF-16 [21]. Furthermore, Parkin, whose gene shares a bi-directional promoter with *PACRG*, has been shown multiple transcriptional regulation activities [22,23,24]. Therefore, these transcription regulators and transcription factors could be attractive targets for deeper functional studies of PACRG.

Three interactors including TUBB2B, TUBGCP4 and CCDC6, which function as structural constituent of cytoskeleton, were also identified (Table 1). PACRG is well known to play important roles in stabilizing microtubule structure via binding with α, β-tubulin [3], whereas TUBGCP4 and CCDC6 were newly found to be potential interaction partners of PACRG in this study. However, these probable interactions require further confirmation through in vitro and in vivo experiments.

Enrichment analysis indicated that the 74 proteins were involved in diverse significant biological processes, among which regulation of nucleotide metabolism and protein metabolism were the first two proportional ones (Table 2). The sublist of regulation of nucleotide metabolism contained 26 proteins, which consisted of most proteins functioning as transcription factor activity, RNA binding and transcription regulator activity (Table 1 and Table 2). The biological process of protein metabolism contained nine members, among which the four proteins HSPD1, HSPA8, HSPA1A and HSPA5 functioned as heat shock protein activity or chaperone activity, and the remaining five proteins served as a ubiquitination pathway enzyme or a ubiquitin receptor (Table 1 and Table 2). Previous investigation showed that PACRG served as an adaptor protein, and facilitated HOIP-dependent linear ubiquitination [14]. Given the fact that genes driven by bidirectional promoters cooperate in common pathways and Parkin is an E3 ubiquitin ligase [25,26], so PACRG may have more functions associated with ubiquitin-dependent protein catabolic process. In this study, five ubiquitination pathway-related proteins, namely MID1, PSMD4, TRIM11, USP39 and USP35, were identified (Table 2). These findings should be helpful for further functional clarification related to PACRG.

Enrichment analysis showed that the most significant enriched biological pathways for the 74 proteins are sphingosine 1-phosphate (S1P) pathway and TRAIL signaling pathway (Figure 5). S1P is a bioactive lipid second messenger that regulates diverse biological processes, and S1P pathway has been implicated in the pathogeneses of autoimmune disease, cancer and other diseases [27,28,29]. Tumor necrosis factor (TNF)-related apoptosis-inducing ligand (TRAIL), a member of the large TNF superfamily, selectively triggers apoptosis in tumor cells, but not normal cells [30,31]. S1P pathway and TRAIL signaling pathway have been extensively investigated as potential targets for treatments of multiple diseases. It has been reported that the bi-directional promoter region of *PACRG* and *Parkin* genes is associated with susceptibility to leprosy and several types of cancer, but the underlying mechanisms are still poorly elucidated [10,13,32,33,34,35,36]. Leprosy is a chronic infectious disease caused by *Mycobacterium leprae*, and clinical manifestations of leprosy are strongly correlated with the host’s immune responses [37]. Given the fact that S1P pathway and TRAIL signaling pathway are associated with the pathogeneses of autoimmune disease and cancer, therefore, further studies on the roles of PACRG related to the two pathways may help to elucidate the association of *PACRG* and *Parkin* polymorphisms with an increased susceptibility to leprosy and cancer.

Overall, in the current study, we have developed a facile approach for soluble expression of PACRG by using the cold-shock vectors, and this method would be very helpful for further research. PACRG consists of an N-terminal divergent region and several conserved regions [3], and functions of these regions are still far from clear. This approach could be applied to express these fragments of PACRG, and identify the interactors of them by combining a pull down assay. Subsequent comparative analysis on the interactors of PACRG and these fragments could facilitate our understanding the different roles of these regions. In addition, a total number of 74 proteins have been identified as potential interaction partners of PACRG in our study, and a higher proportion of the partners function as transcription regulator and transcription factor. Most of the transcription regulators and the transcription factors execute complex physiological functions. For example, transcription factor YY1 (Yin Yang 1) is able to act as both a transcriptional activator and repressor, which depend on various post-translational modifications of YY1 and different co-factors binding with YY1 [38,39,40]. YY1 plays important roles in neuroprotective pathways associated with ischemic damage, Parkinson’s and Alzheimer’s disease, and acts as a tumor suppressor or stimulator [38,41]. Thus, whether and how PACRG interplays with these transcription regulators and transcription factors, such as YY1, would be attractive research topics. Moreover, the identification of five ubiquitination pathway-related proteins, including two ubiquitin ligases MID1 and TRIM11, two deubiquitinating enzymes USP35 and USP39, as well as the proteasome regulatory subunit PSMD4, is also of interest. Given the facts that PACRG has no known catalytic activity and it serves as an adaptor protein to facilitated HOIP-dependent ubiquitination [14], we speculate that PACRG may play an adaptor role in the ubiquitination pathways associated with the five partners. Additional experiments need to be performed to verify this hypothesis. Taken together, the identification of interaction partners provide intriguing candidate targets towards understanding the role of PACRG.

## 4. Materials and Methods

### 4.1. Construction of Expression Plasmids pQE-PACRG, pCold-PACRG and pCold-GST-PACRG

The full-length coding sequence of human *PACRG* (GeneBank NO. AF546872) was amplified by PCR using cDNA reversed from the total RNA of HEK293T cells, and cloned into Tag3B vector. The coding sequence was then cleaved with BamH I and Hind III from Tag3B-PACRG plasmid and subcloned into the expression vectors pQE-80L, pCold II, and pCold-GST in frame, respectively. The entire inserted sequence was verified by nucleotide sequencing. The plasmids were transformed separately into *E. coli* BL21(DE3) strain for effective protein expression. All the three expression plasmids harbor a His_6_ tag in their N-terminal to facilitate Ni^2+^ chelating purification, while pCold-GST-PACRG contains an additional GST tag following its His_6_ tag.

### 4.2. Prokaryotic Expression of pQE-PACRG, pCold-PACRG and pCold-GST-PACRG

Each clone of the three plasmids was grown at 37 °C in LB broth containing 100 mg/L ampicillin under shaking overnight, and the night cultures were diluted 1/50 to 10 mL fresh medium and allowed to grow at 37 °C to middle log phase (OD_600_ between 0.5 to 0.8). Different treatments were carried out for expressions of the three plasmids. Expression of pQE-PACRG was induced with 0.5 mM IPTG for 1 and 2 h at 37 °C, and for 2 h at 20 °C. Expressions of the other two plasmids pCold-PACRG and pCold-GST-PACRG were performed cold-shock treatment prior to IPTG induction, namely, the medium was cooled in ice-water mixture to drop the medium temperature to 15 °C, and kept an additional 30 min. After cold-shock treatment, expressions of pCold-PACRG and pCold-GST-PACRG were induced with several different combinations, including various IPTG concentrations (0.1, 0.2, 0.5, 1 mM) combined with different growth temperature (at 20 °C and 15 °C) for 1, 2, 4, 8 or 12 h (see “results and discussion” for details). The cells were harvested by centrifugation at 5000× *g* for 10 min at 4 °C, and the centrifugated pellets were then collected for detection of expression levels.

### 4.3. Purification of Recombinant Proteins

After detection of the protein expression levels, only the two cold-shock plasmids were subjected to large-scale expression. Each protein was typically expressed in 300 mL LB culture medium with 0.5 mM IPTG induction for 4 h at 15 °C. After centrifugation, the collected cells were resuspended in ice-chilled sonication buffer (PBS supplied with 150 mM sodium chloride (NaCl), 1% Triton X-100, 1 mM phenylmethylsulfonyl fluoride (PMSF), 0.5 mM EDTA, pH 7.0) and sonicated on ice at 300 W (total time 10 min. 3 s on, 5 s off). Then, the lysates were centrifugated at 10,000× *g* for 30 min at 4 °C, and the supernatants were collected for purification. Briefly, after washing with sonication buffer three times, the Ni^2+^-charged resin (Beyotime, Shanghai, China) was mixed with the supernatant. Then, the mixture was incubated at 4 °C with rotation for 2 h. After loading to a 3 mL column, the resin–supernatant reaction was subsequently washed with sonication buffer, 2 mM and 5 mM imidazole. The bound proteins were eluted with 50 mM imidazole (in sonication buffer). While the procedures of GST affinity purification were similar to that of Ni^2+^-charged purification, instead of washing with sonication buffer, and elution with 20 mM glutathione (GSH, in 50 mM Tris-HCl, 150 mM NaCl, 1 mM PMSF, 1 mM EDTA, pH 8.0).

The eluted fractions from Ni^2+^ chelating and GST affinity purification were applied to Sephacryl™ S-200 (GE Healthcare) gel filtration for further purification. A column (1.6 cm × 50 cm) filled with S-200 was equilibrated with working buffer (50 mM Tris-HCl, 150 mM NaCl, 1 mM PMSF, 1 mM EDTA, pH7.0). The fraction (~4 mL) was filtered with a 0.45 μm filter membrane, loaded onto the column, then eluted with the working buffer. PACRG-containing fractions were pooled, concentrated, and stored at −80 °C.

### 4.4. SDS-PAGE and Western Blot

Sodium dodecyl sulfate-polyacrylamide gel electrophoresis (SDS-PAGE) was carried out on 10% polyacrylamide gel, and the gel was stained with Coomassie brilliant blue (CBB) R-250. Western blot (WB) experiments were performed with N-terminal specific PACRG antibody (Ab, 1:1000, Abcam).

### 4.5. His-Tag Pull Down Assay

HEK293T (Human embryonic kidney 293T) cell was previously used for the identification of PACRG-binding proteins with an immunoprecipitation approach [8]. Alternatively, in this study, we performed pull down assays using His_6_-PACRG and His_6_-GST-PACRG as baits to detect interactors of PACRG in HEK293T cell lysates. Briefly, after buffer exchanged with 10 kD ultrafilters, the purified His_6_-PACRG and His_6_-GST-PACRG were coupled separately to Ni^2+^-charged resins for 2 h at 4 °C with rotation. Then, the protein–resin mixtures were incubated with supernatants of HEK293T cell lysates for an additional 2 h. After incubation, the Ni^2+^-charged resins were washed three times with 5 mM imidazole and then boiled in 2 × SDS loading buffer for 5 min. The bound fractions were subjected to SDS-PAGE and CBB staining.

### 4.6. LC-MS/MS Analysis

The bound fractions in Section 4.5 were subjected to 8% SDS-PAGE concentrated gel. After 20 min running, the protein-bromophenol blue bands, which contained the total bound proteins, were excised separately, followed by decolorization in 50% MeOH/50 mM NH_4_HCO_3_, reduction in 25 mM DTT/50 mM NH_4_HCO_3_, alkylation in 55 mM IAA/50 mM NH_4_HCO_3_, and digestion with trypsin at 37 °C overnight. Digestion products were extracted using 0.1% formic acid, 2% acetonitrile. After desalination, peptides were separated by a reverse-phase column (Acclaim PepMap 15 cm × 75 µm, C18, 3 µm, 100 A, Thermo) using acetonitrile gradient containing 0.1% formic acid at a flow rate of 0.3 µL/min for 65 min. Mass spectrometry was achieved using a Thermo Scientific Q Exactive. Mascot generic format (MGF) sample files were then analyzed using Mascot software. Peptides of greater than 95.0% probability were accepted. Each bound protein of His_6_-PACRG and His_6_-GST-PACRG was obtained if either of the following criteria were met: number of detected peptides in the pull down sample ≥2 and no detected peptide in the corresponding control [42]; number of detected peptides in the sample ≥3 and the ratio of peptides number of sample/control ≥2 [43]. Bound proteins of Ni^2+^-charged resin and purified His_6_-GST were used as the control for His_6_-PACRG and His_6_-GST-PACRG, respectively. Bound proteins that were present in both His_6_-PACRG and His_6_-GST-PACRG pull down experiments were accepted as PACRG interaction partners. The obtained proteins were searched against the Universal Protein Resource (UniProt) database. The function classifications of the proteins were performed with FunRich (functional enrichment) software [44].

### 4.7. Cell Culture of HEK293T

HEK293T cells were grown in DMEM medium supplemented with 10% fetal bovine serum and penicillin–streptomycin solution. The cells were lysed for 20 min on ice with cold lysis buffer (same as sonication buffer). The lysates were centrifuged at 20,000× *g* for 10 min at 4 °C, and the supernatant fractions were applied to pull down assay.

## 5. Conclusions

In this work, we developed approaches for the soluble expressions of His_6_-PACRG and His_6_-GST-PACRG using the two cold-shock vectors. The recombinant proteins were purified with Ni^2+^ column, GST affinity chromatography and gel filtration, and the two purified proteins were obtained at yields of ~1.0 mg/L and ~1.5 mg/L of LB culture medium, respectively. Then, we used the purified proteins as baits to isolate and identify functional partners of PACRG. Overall 74 proteins were identified as interaction partners of PACRG, among which 18 proteins function as transcription regulator and transcription factor activities, suggesting that PACRG may play important roles in regulation of gene transcription. In addition, five ones of the 74 interactors are ubiquitination pathway-related proteins, implicating involvement of PACRG in ubiquitin-dependent protein metabolism process. Finally, the N-terminal divergent region of PACRG tended to be insoluble in *E. coli*, and whether this region accounts for the aggregation of PACRG in Lewy bodies may deserve to be further elucidated.

## Figures and Tables

**Table 1:** Function classifications of the *Parkin* co-regulated gene (PACRG) interaction partners.

S. No.	Molecular Function	Gene Names	No. of Genes	*p*-Value
1	Transcription factor activity	UBP1; SMAD4; IRF3; RSRC2; FOXC1; DCP1A; HEXIM1; YY1; BCLAF1; MNX1	10	0.0014
2	Heat shock protein activity	HSPD1; HSPA8	2	0.0048
3	Receptor signaling complex scaffold activity	STAM2; IRS4; SNTB2; PDLIM5; HOMER1	5	0.0080
4	Chaperone activity	HSPA1A; HSPA5	2	0.013
5	RNA binding	DHX15; IGF2BP2; CSTF2; RNPS1; SF1	5	0.013
6	Transcription regulator activity	SMAD3; TRIP4; FUBP1; SNW1; MID1; ZNF24; POLR3C; SAP30BP	8	0.014
7	Intracellular transporter activity	ATG16L1	1	0.015
8	Structural constituent of cytoskeleton	TUBB2B; TUBGCP4; CCDC6	3	0.015
9	ATP binding	ABCD3	1	0.023
10	GTP binding	SEPT9	1	0.030
11	Exonuclease activity	EXD2	1	0.038
12	Enzyme regulator activity	PPP2R1B	1	0.049

**Table 2:** Classifications of biological processes in relation to the PACRG interaction partners.

S. No.	Biological Process	Gene Names	No. of Genes	*p*-Value
1	Regulation of nucleotide metabolism	SMAD3; TRIP4; FUBP1; SNW1; MID1; ZNF24; POLR3C; UBP1; SMAD4; IRF3; FOXC1; DCP1A; HEXIM1; YY1; BCLAF1; MNX1; DHX15; IGF2BP2; CSTF2; RNPS1; SF1; EXD2; HIST2H2BF; ZNF787; ORC2; SETMAR	26	9.86 × 10^−6^
2	Nuclear organization and biogenesis	TMPO	1	0.0039
3	Cell proliferation	RSRC2; SEPT9	2	0.0099
4	Neurotransmitter transport	STXBP1	1	0.015
5	Amino acid and derivative metabolism	ALDH18A1	1	0.015
6	Protein metabolism	HSPD1; HSPA8; HSPA1A; HSPA5; MID1; PSMD4; TRIM11; USP39; USP35	9	0.030
7	Cell surface receptor linked signal transduction	SMAD1	1	0.030
8	Regulation of immune response	HSPD1	1	0.030
9	Regulation of translation	IGF2BP2	1	0.049

## Data Availability

Not applicable.

## Supplementary Materials

The following are available online, Figure S1: Expression profiles of pCold-GST without or with 0.5 mM IPTG induction for various durations at 20 °C, Table S1: List of the 74 potential interaction partners of PACRG.

## References

[1] West A.B., Lockhart P.J., Ofarell C., Farrer M.J. (2003). Identification of a Novel Gene Linked to Parkin via a Bi-directional Promoter. J. Mol. Biol..

[2] Kitada T., Asakawa S., Hattori N., Matsumine H., Yamamura Y., Minoshima S., Yokochi M., Mizuno Y., Shimizu N. (1998). Mutations in the Parkin Gene Cause Autosomal Recessive Juvenile Parkinsonism. Nature.

[3] Ikeda T. (2008). Parkin-co-regulated Gene (PACRG) Product Interacts with Tubulin and Microtubules. FEBS Lett..

[4] Dawe H.R., Farr H., Portman N., Shaw M.K., Gull K. (2005). The Parkin Co-regulated Gene Product, PACRG, is an Evolutionarily Conserved Axonemal Protein That Functions in Outer-doublet Microtubule Morphogenesis. J. Cell Sci..

[5] Mizuno K., Dymek E.E., Smith E.F. (2016). Microtubule Binding Protein PACRG Plays a Role in Regulating Specific ciliary Dyneins during Microtubule Sliding. Cytoskeleton.

[6] Lorenzetti D., Bishop C.E., Justice M.J. (2004). Deletion of the Parkin Coregulated Gene Causes Male Sterility in the *Quaking^viable^* Mouse Mutant. Proc. Natl. Acad. Sci. USA.

[7] Zhao H.Z., Li J., Ma Y.H., Liu W.H. (2018). Research Progress of Parkin Co-regulated Gene. Chin. Pharmacol. Bull..

[8] Imai Y., Soda M., Murakami T., Shoji M., Abe K., Takahashi R. (2003). A Product of the Human Gene Adjacent to *Parkin* is a Component of Lewy Bodies and Suppresses Pael Receptor-induced Cell Death. J. Biol. Chem..

[9] Taylor J.M., Song Y.J., Huang Y., Farrer M.J., Delatycki M.B., Halliday G.M., Lockhart P.J. (2007). Parkin Co-regulated Gene (PACRG) is Regulated by the Ubiquitin–proteasomal System and is Present in the Pathological Features of Parkinsonian Diseases. Neurobio. Dis..

[10] Mira M.T., Alcaïs A., Nguyen V.T., Moraes M.O., Di Flumeri C., Vu H.T., Mai C.P., Nguyen T.H., Nguyen N.B., Pham X.K. (2004). Susceptibility to Leprosy is Associated with *PARK2* and *PACRG*. Nature.

[11] Bragina E.Y., Tiys E.S., Rudko A.A., Ivanisenko V.A., Freidin M.B. (2016). Novel Tuberculosis Susceptibility Candidate Genes Revealed by the Reconstruction and Analysis of Associative Networks. Infect. Genet. Evol..

[12] Ali S., Vollaard A.M., Widjaja S., Surjadi C., van de Vosse E., van Dissel J.T. (2006). PARK2/PACRG Polymorphisms and Susceptibility to Typhoid and Paratyphoid Fever. Clin. Exp. Immunol..

[13] Ichimura K., Mungall A.J., Fiegler H., Pearson D.M., Dunham I., Carter N.P., Peter Collins V. (2006). Small Regions of Overlapping Deletions on 6q26 in Human Astrocytic Tumours Identified Using Chromosome 6 Tile Path Array-CGH. Oncogene.

[14] Meschede J., Šadić M., Furthmann N., Miedema T., Sehr D.A., Müller-Rischart A.K., Bader V., Berlemann L.A., Pilsl A., Schlierf A. (2020). The Parkin-coregulated Gene Product PACRG Promotes TNF Signaling by Stabilizing LUBAC. Sci. Signal..

[15] Li W., Walavalkar N.M., Buchwald W.A., Teves M.E., Zhang L., Liu H., Bilinovich S., Peterson D.L., Strauss J.F., Williams D.C. (2016). Dissecting the Structural Basis of MEIG1 Interaction with PACRG. Sci. Rep..

[16] Khan N., Pelletier D., McAlear T.S., Croteau N., Veyron S., Bayne A.N., Black C., Ichikawa M., Khalifa A.A.Z., Chaaban S. (2021). Crystal Structure of Human PACRG in Complex with MEIG1 Reveals Roles in Axoneme Formation and Tubulin binding. Structure.

[17] Qing G., Ma L.C., Khorchid A., Swapna G.V., Mal T.K., Takayama M.M., Xia B., Phadtare S., Ke H., Acton T. (2004). Cold-shock Induced High-yield Protein Production in *Escherichia coli*. Nat. Biotechnol..

[18] Sugiki T., Fujiwara T., Kojima C. (2017). Cold-Shock Expression System in *E. coli* for Protein NMR Studies. Methods Mol. Biol..

[19] Hayashi K., Kojima C. (2008). pCold-GST Vector: A Novel Cold-shock Vector Containing GST Tag for Soluble Protein Production. Protein. Expr. Purif..

[20] Lee D., Han S., Woo S., Lee H.W., Sun H., Kim W. (2014). Enhanced Expression and Purification of Inositol 1,4,5-trisphosphate 3-kinase A through Use of the pCold1-GST Vector and a C-terminal Hexahistidine Tag in *Escherichia coli*. Protein. Expr. Purif..

[21] Loucks C.M., Bialas N.J., Dekkers M.P., Walker D.S., Grundy L.J., Li C., Inglis P.N., Kida K., Schafer W.R., Blacque O.E. (2016). PACRG, a Protein Linked to Ciliary Motility, Mediates Cellular Signaling. Mol. Biol. Cell..

[22] Shires S.E., Quiles J.M., Najor R.H., Leon L.J., Cortez M.Q., Lampert M.A., Mark A., Gustafsson Å.B. (2020). Nuclear Parkin Activates the ERRα Transcriptional Program and Drives Widespread Changes in Gene Expression Following Hypoxia. Sci. Rep..

[23] Nezich C.L., Wang C., Fogel A.I., Youle R.J. (2015). MiT/TFE Transcription Factors are Activated during Mitophagy Downstream of Parkin and Atg5. J. Cell Biol..

[24] Ren Y., Jiang H., Ma D., Nakaso K., Feng J. (2011). Parkin Degrades Estrogen-related Receptors to Limit the Expression of Monoamine Oxidases. Hum. Mol. Genet..

[25] Li Y.Y., Yu H., Guo Z.M., Guo T.Q., Tu K., Li Y.X. (2006). Systematic Analysis of Head-to-head Gene Organization: Evolutionary Conservation and Potential Biological Relevance. PLoS Comput. Biol..

[26] Shimura H., Hattori N., Kubo S., Mizuno Y., Asakawa S., Minoshima S., Shimizu N., Iwai K., Chiba T., Tanaka K. (2000). Familial Parkinson Disease Gene Product, Parkin, is a Ubiquitin-protein Ligase. Nat. Genet..

[27] Tsai H.C., Han M.H. (2016). Sphingosine-1-phosphate (S1P) and S1P Signaling Pathway: Therapeutic Targets in Autoimmunity and Inflammation. Drugs.

[28] Grbčić P., Sedić M. (2020). Sphingosine 1-phosphate Signaling and Metabolism in Chemoprevention and Chemoresistance in Colon Cancer. Molecules.

[29] Gomez-Larrauri A., Presa N., Dominguez-Herrera A., Ouro A., Trueba M., Gomez-Muñoz A. (2020). Role of Bioactive Sphingolipids in Physiology and Pathology. Essays Biochem..

[30] Woo S.M., Kwon T.K. (2019). E3 Ubiquitin Ligases and Deubiquitinases as Modulators of TRAIL-mediated Extrinsic Apoptotic Signaling Pathway. BMB Rep..

[31] Stöhr D., Jeltsch A., Rehm M. (2020). TRAIL Receptor Signaling: From the Basics of Canonical Signal Transduction Toward Its Entanglement with ER Stress and the Unfolded Protein Response. Int. Rev. Cell Mol. Biol..

[32] Mazini P.S., Alves H.V., Reis P.G., Lopes A.P., Sell A.M., Santos-Rosa M., Visentainer J.E., Rodrigues-Santos P. (2016). Gene Association with Leprosy: A Review of Published Data. Front. Immunol..

[33] Leturiondo A.L., Noronha A.B., Mendonça C.Y.R., Ferreira C.O., Alvarado-Arnez L.E., Manta F.S.N., Bezerra O.C.L., Carvalho E.F., Moraes M.O., Rodrigues F.D.C. (2020). Association of NOD2 and IFNG Single Nucleotide Polymorphisms with Leprosy in the Amazon Ethnic Admixed Population. PLoS Negl. Trop. Dis..

[34] Agirre X., Román-Gómez J., Vázquez I., Jiménez-Velasco A., Garate L., Montiel-Duarte C., Artieda P., Cordeu L., Lahortiga I., Calasanz M.J. (2006). Abnormal Methylation of the Common *PARK2* and *PACRG* Promoter is Associated with Downregulation of Gene Expression in Acute Lymphoblastic Leukemia and Chronic Myeloid Leukemia. Int. J. Cancer.

[35] Toma M.I., Wuttig D., Kaiser S., Herr A., Weber T., Zastrow S., Koch R., Meinhardt M., Baretton G.B., Wirth M.P. (2013). *PARK2* and *PACRG* are Commonly Downregulated in Clear-cell Renal Cell Carcinoma and are Associated with Aggressive Disease and Poor Clinical Outcome. Genes Chromosomes Cancer.

[36] Han B., Yang X., Zhang P., Zhang Y., Tu Y., He Z., Li Y., Yuan J., Dong Y., Hosseini D.K. (2020). DNA Methylation Biomarkers for Nasopharyngeal Carcinoma. PLoS ONE.

[37] Froes Jr L.A.R., Trindade M.A.B., Sotto M.N. (2020). Immunology of Leprosy. Int. Rev. Immunol..

[38] Verheul T.C.J., van Hijfte L., Perenthaler E., Barakat T.S. (2020). The Why of YY1: Mechanisms of Transcriptional Regulation by Yin Yang 1. Front. Cell Dev. Biol..

[39] Yao Y.L., Yang W.M., Seto E. (2001). Regulation of Transcription Factor YY1 by Acetylation and Deacetylation. Mol. Cell. Biol..

[40] Gordon S., Akopyan G., Garban H., Bonavida B. (2006). Transcription Factor YY1: Structure, Function, and Therapeutic Implications in Cancer Biology. Oncogene.

[41] Khachigian L.M. (2018). The Yin and Yang of YY1 in Tumor Growth and Suppression. Int. J. Cancer.

[42] Krajewska J., Arent Z., Zolkiewski M., Kędzierska-Mieszkowska S. (2018). Isolation and Identification of Putative Protein Substrates of the AAA+ Molecular Chaperone ClpB from the Pathogenic Spirochaete *Leptospira interrogans*. Int. J. Mol. Sci..

[43] Zhang P.P., Yan H., Yuan Y.F., Feng Y.Q. (2017). Searching for Dysbindin-1 Interacting Proteins in Mouse Testis by GST Pull-down and Mass Spectrometry. Chin. J. Biochem. Mol. Biol..

[44] Pathan M., Keerthikumar S., Ang C.S., Gangoda L., Quek C.Y.J., Williamson N.A., Mouradov D., Sieber O.M., Simpson R.J., Salim A. (2015). FunRich: An Open Access Standalone Functional Enrichment and Interaction Network Analysis Tool. Proteomics.

