# Supplementary material for: Functional Expression, Purification and Identification of Interaction Partners of PACRG
Source: Molecules. 2021 Apr 16;26(8):2308. doi: 10.3390/molecules26082308 (PMC8074078; doi:10.3390/molecules26082308)
Supplement: Supplementary file 1 [file molecules-26-02308-s001.zip › molecules-1150294-supplementary.pdf]

Table S1. List of the 74 potential interaction partners of PACRG

S. No.	Uniprot ID	Protein names	Gene names
1	Q9NZI7	Upstream-binding protein 1	UBP1
2	Q13485	Mothers against decapentaplegic homolog 4	SMAD4
3	Q14653	Interferon regulatory factor 3 (IRF-3)	IRF3
4	Q7L4I2	Arginine/serine-rich coiled-coil protein 2	RSRC2
5	Q12948	Forkhead box protein C1	FOXC1
6	Q9NPI6	mRNA-decapping enzyme 1A	DCP1A
7	O94992	Protein HEXIM1	HEXIM1
8	P25490	Transcriptional repressor protein YY1	YY1
9	Q9NYF8	Bcl-2-associated transcription factor 1	BCLAF1
10	P50219	Motor neuron and pancreas homeobox protein 1	MNX1
11	P10809	60 kDa heat shock protein, mitochondrial	HSPD1
12	P11142	Heat shock cognate 71 kDa protein	HSPA8
13	O75886	Signal transducing adapter molecule 2	STAM2
14	O14654	Insulin receptor substrate 4 (IRS-4)	IRS4
15	Q13425	Beta-2-syntrophin	SNTB2
16	Q96HC4	PDZ and LIM domain protein 5	PDLIM5
17	Q86YM7	Homer protein homolog 1 (Homer-1)	HOMER1
18	P0DMV8	Heat shock 70 kDa protein 1A (HSP70-1)	HSPA1A
19	P11021	Endoplasmic reticulum chaperone BiP	HSPA5
20	O43143	Pre-mRNA-splicing factor ATP-dependent RNA helicase DHX15	DHX15
21	Q9Y6M1	Insulin-like growth factor 2 mRNA-binding protein 2	IGF2BP2
22	P33240	Cleavage stimulation factor subunit 2	CSTF2
23	Q15287	RNA-binding protein with serine-rich domain 1	RNPS1
24	Q15637	Splicing factor 1	SF1
25	P84022	Mothers against decapentaplegic homolog 3	SMAD3
26	Q15650	Activating signal cointegrator 1 (ASC-1)	TRIP4
27	Q96AE4	Far upstream element-binding protein 1	FUBP1
28	Q13573	SNW domain-containing protein 1	SNW1
29	O15344	E3 ubiquitin-protein ligase Midline-1	MID1
30	P17028	Zinc finger protein 24	ZNF24
31	Q9BUI4	DNA-directed RNA polymerase III subunit RPC3	POLR3C
32	Q9UHR5	SAP30-binding protein	SAP30BP
33	Q676U5	Autophagy-related protein 16-1 (APG16-like 1)	ATG16L1;
34	Q9BVA1	Tubulin beta-2B chain	TUBB2B
35	Q9UGJ1	Gamma-tubulin complex component 4 (GCP-4)	TUBGCP4
36	Q16204	Coiled-coil domain-containing protein 6	CCDC6
37	P28288	ATP-binding cassette sub-family D member 3	ABCD3
38	Q9UHD8	Septin-9	SEPT9
39	Q9NVH0	Exonuclease 3'-5' domain-containing protein 2	EXD2
40	P30154	Serine/threonine-protein phosphatase 2A 65 kDa regulatory subunit A beta isoform	PPP2R1B
41	Q86TJ2	Transcriptional adapter 2-beta	TADA2B
42	P54886	Delta-1-pyrroline-5-carboxylate synthase (P5CS)	ALDH18A1
43	P55036	26S proteasome non-ATPase regulatory subunit 4	PSMD4
44	Q96F44	E3 ubiquitin-protein ligase TRIM11	TRIM11
45	Q53GS9	U4/U6.U5 tri-snRNP-associated protein 2	USP39
46	P49761	Dual specificity protein kinase CLK3	CLK3

47	Q15797	Mothers against decapentaplegic homolog 1	SMAD1
48	O95831	Apoptosis-inducing factor 1, mitochondrial	AIFM1
49	Q9P2H5	Ubiquitin carboxyl-terminal hydrolase 35	USP35;
50	Q15691	Microtubule-associated protein RP/EB family member 1	MAPRE1
51	Q9P258	Protein RCC2	RCC2
52	P80303	Nucleobindin-2 (DNA-binding protein NEFA)	NUCB2
53	Q02818	Nucleobindin-1 (CALNUC)	NUCB1
54	O96013	Serine/threonine-protein kinase PAK 4	PAK4
55	Q5T0N5	Formin-binding protein 1-like	FNBP1L
56	Q6P597	Kinesin light chain 3	KLC3
57	Q5QNW6	Histone H2B type 2-F	HIST2H2BF
58	Q6DD87	Zinc finger protein 787	ZNF787
59	Q12899	Tripartite motif-containing protein 26	TRIM26
60	Q13416	Origin recognition complex subunit 2	ORC2
61	P42167	Lamina-associated polypeptide 2, isoforms beta/gamma	LAP2B
62	P48651	Phosphatidylserine synthase 1 (PSS-1)	PTDSS1
63	Q9NSK0	Kinesin light chain 4 (KLC 4)	KLC4
64	P61764	Syntaxin-binding protein 1	STXBP1
65	O14531	Dihydropyrimidinase-related protein 4 (DRP-4)	DPYSL4
66	P10636	Microtubule-associated protein tau	MAPT
67	Q8IZ69	tRNA (uracil-5-)-methyltransferase homolog A	TRMT2A
68	Q4G0J3	La-related protein 7	LARP7
69	Q8IVM0	Coiled-coil domain-containing protein 50	CCDC50;
70	Q53H47	Histone-lysine N-methyltransferase SETMAR	SETMAR
71	P42166	Lamina-associated polypeptide 2, isoform alpha	TMPO;
72	Q9UG63	ATP-binding cassette sub-family F member 2	ABCF2
73	Q96RS6	NudC domain-containing protein 1	NUDCD1;
74	Q9NVN8	Guanine nucleotide-binding protein-like 3-like protein	GNL3L

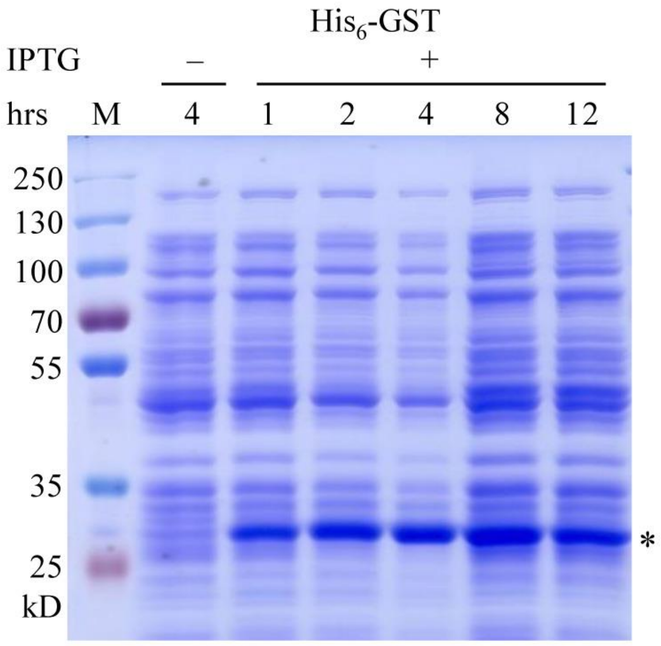

Figure S1. Expression profiles of pCold-GST without or with 0.5 mM IPTG induction for various durations at 20 ℃. * denote His_6-GST. M, protein marker.
